# The role of leadership in job satisfaction and turnover intention among Navy nurses: a cross-sectional study in Greece

**DOI:** 10.3389/frhs.2025.1712270

**Published:** 2026-01-16

**Authors:** Stefanos Karakolias, Evangelia Schiza

**Affiliations:** 1 Department of Nursing, Democritus University of Thrace, Alexandroupolis, Greece; 2 Postgraduate Program in Health Care Units Management, Hellenic Open University, Patras, Greece; 3 Athens Naval Hospital, Athens, Greece

**Keywords:** cross-sectional study, Greece, job satisfaction, leadership, navy nurses, supervision, turnover intention

## Abstract

**Background:**

Leadership underpins nurses' interrelated satisfaction and retention, particularly in military healthcare settings marked by strict hierarchies and high operational demands. In Greece, Navy nurses are integral to military healthcare, yet evidence on their job satisfaction and turnover intentions remains limited. This study offers an initial examination of these factors to inform retention strategies and sustain clinical workforce stability.

**Methods:**

A cross-sectional survey was conducted between January and March 2025 among active-duty Hellenic Navy nurse officers, primarily serving at the Athens Naval Hospital. Data were collected using an anonymous questionnaire incorporating the Job Satisfaction Survey (JSS) and turnover intention items. Analyses employed descriptive statistics and bivariate tests.

**Results:**

Sixty nurse officers participated (response rate: 53.6%). While respondents reported satisfaction with supervision (68.3%) and coworker relationships (31.7%), there was widespread dissatisfaction with extrinsic factors: 90.0% with pay, 85.0% with promotion opportunities, and 83.3% with fringe benefits. Overall, 53.3% of nurses reported low job satisfaction. Turnover intentions were high: 46.7% considered transitioning to civilian nursing, and 31.7% intended to leave both the Navy and the profession. Job satisfaction related to the nature of work (*r* = 0.36, *p* < 0.05) and communication (*r* = −0.33, *p* < 0.05) significantly correlated with turnover intentions.

**Conclusion:**

Leadership quality, as reflected in supportive supervision, is a key protective factor against job dissatisfaction and turnover intention among Hellenic Navy nurse officers. Strengthening supervisory practices and leadership development, alongside reforms addressing extrinsic rewards and communication, is essential to enhance retention and sustain a resilient military nursing workforce.

## 1 Introduction

Job satisfaction is a multifaceted construct reflecting the extent to which employees experience positive feelings about their work and perceive it as meaningful in their lives (1, 2). In nursing, job satisfaction is closely linked to professional well-being, quality of care, and workforce stability. Turnover intention, in contrast, refers to an employee's conscious and deliberate decision to leave their current job or organization and represents a psychological state that precedes actual turnover behavior (3). A substantial body of evidence consistently demonstrates a negative association between job satisfaction and turnover intention among nurses, underscoring the importance of understanding the factors that promote satisfaction and retention (4).

Leadership plays a central role in shaping nurses' work experiences and attitudes. It is commonly understood as the process through which individuals use knowledge, skills, and behaviors to guide others toward shared goals (5). Leadership influences motivation, communication, professional autonomy, and perceptions of organizational support, thereby directly affecting job satisfaction and turnover intentions. Contemporary research highlights that effective leadership practices—such as transformational and authentic leadership—are associated with higher job satisfaction and lower turnover intention, whereas ineffective or toxic leadership styles are linked to increased stress, dissatisfaction, and attrition (6–10).

Job satisfaction among nurses is influenced by a complex interplay of organizational conditions, interpersonal relationships, individual characteristics, and leadership practices. Herzberg's two-factor theory remains a useful framework for understanding these dynamics, distinguishing intrinsic motivators—such as meaningful work, recognition, and professional growth—from extrinsic or hygiene factors, including supervision, working conditions, pay, promotion opportunities, and job security (11). While intrinsic aspects of nursing often support professional commitment, dissatisfaction with extrinsic conditions has been consistently associated with increased turnover intention and intention to leave the profession (12–15). Recent evidence further emphasizes the role of work meaningfulness, supportive practice environments, and career alignment in shaping nurses' satisfaction and retention decisions (1, 2, 13–15).

Contemporary healthcare systems are characterized by increasing clinical complexity, staffing shortages, administrative burden, and heightened performance expectations. These pressures intensify nurses’ workload and psychological demands, increasing vulnerability to burnout—a syndrome characterized by emotional exhaustion, depersonalization, and reduced personal accomplishment (16, 17). Burnout has been extensively linked to adverse patient outcomes, including compromised quality of care, patient safety incidents, and reduced patient satisfaction (18, 19). Importantly, burnout also has significant nurse-related consequences. Evidence consistently demonstrates strong associations between burnout, reduced job satisfaction, increased turnover intention, and decisions to leave the nursing profession altogether, highlighting burnout as a critical threat to workforce sustainability (11, 12, 18, 19).

These challenges are particularly salient in military healthcare settings. Military nurses provide care in highly structured and demanding environments and are often exposed to additional psychological, ethical, and operational stressors. Such conditions may amplify the effects of organizational factors, leadership practices, and burnout on job satisfaction and turnover intention (16, 20). Despite their essential role in maintaining operational readiness and continuity of care, empirical research examining job satisfaction and turnover intention among military nursing populations remains limited.

In Greece, military nursing has a longstanding tradition, with Navy nurses trained at the Military Nursing Academy (SAN) and primarily employed at the Athens Naval Hospital (NNA), the main healthcare facility of the Hellenic Navy. While previous studies have examined job satisfaction and work-related stress among Army nurses and civilian nurses (21), no studies have specifically explored job satisfaction, leadership, and turnover intention among Navy nurses.

Accordingly, this study aims to address this gap. The primary objective was to investigate levels of job satisfaction and turnover intention among Greek Navy nurses. Secondary objectives were to explore the relationships between specific facets of job satisfaction and turnover intention and to examine the influence of leadership—particularly supervision—on these outcomes.

## 2 Methods

### 2.1 Study design and reporting

This cross-sectional study was designed and reported in accordance with the Strengthening the Reporting of Observational Studies in Epidemiology (STROBE) guidelines (22), and a completed checklist is provided as Supplementary Table S1. Data collection took place between January and March 2025, with all data obtained at a single time point. As a cross-sectional design was used, there was no exposure period or follow-up.

### 2.2 Study setting

The study was conducted at the NNA, the main healthcare facility of the Hellenic Navy and the primary employer of active-duty Navy nurse officers. The NNA provides secondary-level inpatient care, outpatient clinical services, and emergency care. Nurses seconded to other settings (e.g., warships) were also eligible to participate; however, recruitment and data collection were coordinated centrally through the NNA. Accordingly, the study is considered a single-center investigation. Detailed institutional characteristics such as the number of beds, specific service lines, and total workforce size were not disclosed for the purposes of this study due to organizational confidentiality restrictions.

### 2.3 Participants

All active-duty Navy nurse officers who had graduated from the SAN and were serving within the Hellenic Navy at the time of the study were eligible to participate (total population: 112). The research team approached all eligible nurses across departments and shifts to minimize selection bias, and participation was voluntary. Nurses who declined participation most commonly cited workload constraints or long-term leave as reasons for non-participation.

This study deliberately focused on commissioned Navy nurse officers, all of whom are graduates of the SAN and hold university-level nursing degrees. Within Greek military healthcare settings, nursing personnel may also include other categories, such as vocationally trained nurses or licensed practical nurses; however, these groups were outside the scope of the present study. This focus was chosen to ensure a homogeneous study population and to enhance the interpretability of leadership, job satisfaction, and turnover intention within this specific upper-level professional cohort.

### 2.4 Data collection and instruments

Data were collected using a paper-based, anonymous, self-administered questionnaire consisting of three sections: demographic and professional characteristics; job satisfaction; and turnover intention.

Job satisfaction was measured with the 36-item Job Satisfaction Survey (JSS), developed by Spector (1985) (23), assessing nine domains (pay, promotion, supervision, fringe benefits, contingent rewards, operating procedures, coworkers, nature of work, and communication) on a 6-point Likert scale ranging from “strongly disagree” to “strongly agree.” Higher scores indicated greater satisfaction. Reverse-scored items were recoded before computing domain and total scores.

Turnover intention was assessed using items adapted from established nursing turnover intention measures (24, 25), also rated on a 6-point Likert scale for consistency. Predictor variables included demographic and professional characteristics (age, gender, marital status, number of children, education level, rank, and years of service) and perceived leadership quality (JSS supervision subscale). Because the study was descriptive, no confounders or effect modifiers were modeled. All participants received the same instruments and instructions, ensuring full comparability across subgroups.

### 2.5 Bias and study size

Multiple procedures minimized potential bias. To reduce selection bias, all eligible nurses were invited to participate regardless of shift. To reduce information and social desirability bias, questionnaires were anonymous and confidentiality was emphasized. Measurement bias was minimized by administering identical instruments under uniform conditions. Because there was no follow-up, attrition bias was not applicable.

Because the population was finite and clearly defined, and the study exploratory in nature, no *a priori* power calculation was performed and a census-like sampling approach was adopted.

### 2.6 Statistical analysis

Quantitative variables were treated as continuous and summarized using means and standard deviations. Job satisfaction and turnover intention scores were analyzed in their original continuous form, and no continuous variables were categorized. Pearson correlation coefficients examined associations between satisfaction dimensions and turnover intention. Group comparisons were performed using *t*-tests for two independent groups and one-way ANOVA for comparisons across multiple demographic subgroups.

In the event of missing data, analyses were planned to use pairwise deletion, whereby cases with missing responses on specific items would be excluded only from the relevant analyses. Statistical significance was set at *p* < 0.05, and analyses were conducted using SPSS version 26.0.

### 2.7 Ethical considerations

All participants received written information about the study, confidentiality protections, and their right to withdraw at any time, and provided written informed consent. Ethical approval was granted by the Ethics Committee of the Faculty of Social Sciences of the Hellenic Open University (Approval No. 154250, November 19, 2024).

## 3 Results

Of the 112 eligible active-duty Navy nurse officers invited to participate, 60 returned completed questionnaires, yielding a response rate of 53.6%. All returned questionnaires were complete, and no missing data were identified.

Table 1 presents the demographic and professional characteristics of the study participants. The sample was predominantly female (86.7%). The age distribution shows that the majority of participants (40%) were between 41 and 50 years old, followed by 25% in the 31–40 age group, and 21.7% 51 years or older. Regarding family status, 70% of the participants were married. The vast majority (78.3%) worked at the NNA, with smaller percentages in other naval services. In terms of work departments, 40% were in the pathology sector (i.e., general medical and subspecialty wards such as internal medicine, cardiology, and pulmonology), 25% in the surgery sector, 10% in the intensive care unit, and 3.3% in administrative positions. The participants had varying years of service, with the largest group (18.3%) having 31 years or more of experience. Most participants (65%) worked on a rolling shift schedule, while 30% worked morning shifts and 5% worked 24-h shifts. Regarding education, it is important to contextualize the educational qualifications within the Greek system. The university-level nursing degree (BSc) is a four-year undergraduate program providing foundational professional qualification. The postgraduate degrees mentioned (e.g., Master's) represent an additional one to two years of specialized study in a specific clinical or administrative field, indicating advanced expertise. A PhD is the highest academic qualification, involving several years of original research. In this study, all participants held at least a BSc, and a significant portion (43.3%) had pursued advanced education, with 40% holding postgraduate degrees and 3.3% a PhD, reflecting a highly educated and specialized cohort.

**Table 1:** Demographic and professional features of the sample.

Features	Participants (*n* = 60)	*N*	%
Gender	Female	52	86.7
	Male	8	13.3
Age (years)	20–30	8	13.3
	31–40	15	25.0
	41–50	24	40.0
	≥51	13	21.7
Marital status	Married	42	70.0
	Single	18	30.0
Employer	Athens Naval Hospital	47	78.3
	Hellenic Navy General Staff	2	3.3
	Warship	7	11.7
	Military Nursing Academy	4	6.7
Work department	Pathology sector^a^	24	40.0
	Surgery sector	15	25.0
	Intensive care unit	6	10.0
	Administration	2	3.3
	Other	13	21.7
Years of service	1–5	7	11.7
	6–10	8	13.3
	11–15	6	10.0
	16–20	10	16.7
	21–25	10	16.7
	26–30	8	13.3
	≥31	11	18.3
Work schedule	Morning shift	18	30.0
	Rolling shift	39	65.0
	24-h shift	3	5.0
Education	BSc	34	56.7
	Postgraduate	24	40.0
	PhD	2	3.3

a Refers to non-surgical medical departments, including internal medicine, cardiology, and pulmonology wards.

The results presented in Table 2 provide a comprehensive overview of job satisfaction within the study sample of Hellenic Navy nurse officers across multiple facets. The highest levels of satisfaction were observed in relation to supervision, with a mean score (*M*) of 18.3 and standard deviation (SD) of 4.7, where 68.3% of respondents reported being satisfied and only 11.7% were dissatisfied. Coworker relationships were also evaluated positively, with a mean score of 15.2 (SD = 3.0); although 55.0% of participants were ambivalent, nearly one-third (31.7%) expressed satisfaction, and only 13.3% expressed dissatisfaction. The nature of work dimension received moderate evaluations (*M* = 14.4, SD = 3.9), with responses distributed across ambivalence (46.7%), satisfaction (28.3%), and dissatisfaction (25.0%). Communication was assessed less favorably (*M* = 13.4, SD = 3.1), with 36.7% expressing dissatisfaction, 45.0% ambivalence, and 18.3% satisfaction.

**Table 2:** Job satisfaction levels by JSS facet subscale.

Code	JSS facet subscale	Mean (M)	Standard deviation (SD)	Dissatisfied^a^		Ambivalent^b^		Satisfied^c^	
				*N*	%	*N*	%	*N*	%
S1	Supervision	18.3	4.7	7	11.7	12	20.0	41	68.3
S2	Coworkers	15.2	3.0	8	13.3	33	55.0	19	31.7
S3	Nature of work	14.4	3.9	15	25.0	28	46.7	17	28.3
S4	Communication	13.4	3.1	22	36.7	27	45.0	11	18.3
S5	Operating conditions	9.9	3.0	48	80.0	12	20.0	0	0.0
S6	Contingent rewards	9.7	3.7	45	75.0	14	23.3	1	1.7
S7	Promotion	9.0	3.0	51	85.0	9	15.0	0	0.0
S8	Fringe benefits	8.9	3.3	50	83.3	9	15.0	1	1.7
S9	Pay	8.1	3.4	54	90.0	4	6.7	2	3.3
S10	Total satisfaction	106.9	19.4	32	53.3	26	43.3	2	3.3

a Score range 4–12 for items 1–9 and 36–108 for item 10.

b Score range 12–16 for items 1–9 and 108–144 for item 10.

c Score range 16–24 for items 1–9 and 144–216 for item 10.

In stark contrast, the lowest levels of satisfaction were evident in facets related to organizational policies and rewards. Operating conditions (*M* = 9.9, SD = 3.0), contingent rewards (*M* = 9.7, SD = 3.7), promotion opportunities (*M* = 9.0, SD = 3.0), fringe benefits (*M* = 8.9, SD = 3.3), and pay (*M* = 8.1, SD = 3.4) all elicited substantial dissatisfaction, with over three-quarters of respondents dissatisfied in each category. Dissatisfaction was particularly pronounced for pay (90.0%) and promotion opportunities (85.0%). Overall job satisfaction was low, as reflected in the total JSS mean score of 106.9 (SD = 19.4), just below the ambivalence threshold. More than half of the respondents (53.3%) reported overall dissatisfaction, 43.3% were ambivalent, and only 3.3% reported satisfaction.

Table 3 summarizes the findings on turnover intention among the nurse officers who participated in this study. It should be noted that turnover intention responses, originally captured on a 6-point Likert scale, were dichotomized into two groups: “Disagree” (scores 1–3) and “Agree” (scores 4–6). This approach was chosen to enhance clarity and provide a straightforward interpretation of the overall sentiment regarding turnover, clearly distinguishing between participants who leaned towards staying vs. those who leaned towards leaving. In this context, the majority of participants expressed a strong preference to remain in their current nursing roles within the Navy, with 88.3% disagreeing with the statement that they wished to change their specialty (*M* = 1.6, SD = 1.2). Despite this, notable turnover intentions were observed. Nearly half of the respondents (46.7%) indicated that they would consider pursuing nursing as a civilian career. Additionally, approximately one-third (31.7%) reported intentions to leave both the Navy and the nursing profession entirely.

**Table 3:** Combined turnover intention.

Code	Turnover intention statement	Mean (*M*)	Standard deviation (SD)	Disagree (1–3)		Agree (4–6)	
				N	%	N	%
T1	I would like to pursue nursing as a civilian career, outside the Navy.	3.3	1.9	32	53.3	28	46.7
T2	I wish to remain in the Navy, but I would like to change my specialty (other than nursing)	1.6	1.2	53	88.3	7	11.7
T3	I intend to leave both the Navy and the nursing profession.	2.9	1.5	41	68.3	19	31.7

Finally, the analysis indicated that demographic and professional characteristics had no consistent significant associations with job satisfaction or turnover intentions, as evidenced by the ANOVA and *t*-test results reported in Supplementary Tables S2–S9. In contrast, strong and meaningful relationships emerged between specific facets of job satisfaction and turnover intention. As shown in Table 4, turnover intention was most closely associated with satisfaction related to the nature of work (S3) and communication (S4). Higher satisfaction with the nature of work and effective communication within the organization was associated with a reduced desire to change Navy specialties (T2). Conversely, lower satisfaction with the nature of work increased the likelihood of intending to leave both the Navy and the nursing profession entirely (T3). In contrast, intentions to pursue a civilian nursing career (T1) demonstrated no strong associations with any job satisfaction dimensions.

**Table 4:** Correlation matrix.

Code	S9	S7	S1	S8	S6	S5	S2	S3	S4	S10	T1	T2	T3
T1	0.06	−0.16	−0.25	−0.20	−0.11	−0.23	−0.17	−0.08	−0.03	−0.21	1.00		
T2	−0.22	0.00	−0.03	0.01	−0.20	−0.15	−0.03	−**0.44 (*p*** **<** **0.05)**	**−0.33 (*p*** **<** **0.05)**	**−**0.25	−0.02	1.00	
T3	−0.17	0.03	0.20	−0.19	−0.03	0.12	0.12	**0.36 (*p*** **<** **0.05)**	0.17	0.12	−0.14	−**0.29 (*p*** **<** **0.05)**	1.00

T1: I would like to pursue nursing as a civilian career, outside the Navy.

T2: I wish to remain in the Navy, but I would like to change my specialty (other than nursing).

T3: I intend to leave both the Navy and the nursing profession.

S1: Supervision.

S2: Coworkers.

S3: Nature of work.

S4: Communication.

S5: Operating conditions.

S6: Contingent rewards.

S7: Promotion.

S8: Fringe benefits.

S9: Pay. Boldface values denote statistically significant correlations at the *p* < 0.05 level.

## 4 Discussion

### 4.1 Overview of key findings

The findings of this study highlight a clear distinction between intrinsic and extrinsic determinants of job satisfaction among Greek Navy nurses. Interpersonal and intrinsic aspects of the job—particularly leadership quality, meaningful work, and collegial relationships—emerged as major sources of satisfaction and reinforced professional engagement. In contrast, extrinsic factors such as workload, bureaucratic constraints, salary, promotion opportunities, and fringe benefits were consistently reported as dissatisfying and appear to be important contributors to turnover intention. These findings are consistent with previous evidence indicating that intrinsic motivation and supportive leadership promote job satisfaction, whereas inadequate organizational rewards and structural barriers undermine retention (26, 27).

Importantly, these results should be interpreted within the specific context of a military healthcare setting. The Hellenic Navy is characterized by a highly structured and hierarchical organizational environment, with clearly defined command chains, standardized roles, and limited flexibility in areas such as remuneration, promotion pathways, and career mobility. Such structural characteristics may help explain why dissatisfaction was more pronounced in extrinsic domains, while interpersonal and leadership-related factors remained relatively strong sources of satisfaction. In this context, proximal leadership—particularly supervision—may play a disproportionately important role in shaping nurses' daily work experiences and professional commitment.

The strong positive association between supervision and job satisfaction underscores the importance of leadership behaviors that foster trust, clarity, and professional support. This finding aligns with international literature from both military and civilian nursing contexts, which consistently demonstrates that effective leadership enhances job satisfaction and reduces turnover intention, particularly in demanding healthcare environments (28, 29). Conversely, dysfunctional or toxic leadership styles have been linked to increased stress, absenteeism, and elevated turnover, further highlighting leadership quality as a critical determinant of workforce stability (30, 31).

Job satisfaction was also closely related to nurses' intentions to remain in the Hellenic Navy. Higher satisfaction with supervision, the nature of work, and communication was associated with a lower likelihood of turnover intention, reflecting patterns widely reported in broader nursing research (32). As noted in previous studies, the association between job satisfaction and intention to remain in the profession is well established and not unexpected. Evidence from diverse nursing populations demonstrates that satisfied nurses are more likely to remain in their roles and in the profession overall (33, 34). The present findings extend this well-documented relationship to the context of Navy nurse officers, suggesting that similar mechanisms linking satisfaction and retention operate within military healthcare systems.

The analyses did not reveal statistically significant differences in job satisfaction or turnover intention across service lines or work departments. This suggests that the challenges associated with extrinsic dissatisfaction and the protective role of leadership may be experienced relatively uniformly across clinical areas within the Navy setting, rather than being confined to specific departments. Such uniformity may reflect the centralized organizational structure and standardized working conditions characteristic of military healthcare environments.

Another noteworthy finding was that demographic characteristics were not consistently associated with job satisfaction or turnover intention in this sample. This pattern aligns with evidence suggesting that organizational culture, leadership, and working conditions exert a stronger influence on nurses' well-being and retention than individual demographic attributes (35, 36). These findings further underscore the importance of system-level, rather than individually targeted, strategies to improve satisfaction and retention (37).

Nevertheless, these results should be interpreted in light of contradictory findings in the literature. Several studies conducted in civilian nursing populations have reported that younger nurses or those with fewer years of professional experience may be less likely to intend to leave compared with older, more experienced nurses (34, 38). Such discrepancies may reflect contextual differences between civilian and military healthcare systems, variations in career trajectories, or cohort-specific expectations regarding stability, advancement, and professional identity.

Overall, the results indicate that Greek Navy nurses report moderate job satisfaction, with dissatisfaction in extrinsic factors showing the strongest relationship with turnover intention. While the cross-sectional design precludes causal inference and the single-site military setting may limit generalizability, the consistency of these findings with evidence from both military and civilian contexts supports their credibility. The prominence of leadership and organizational conditions as determinants of satisfaction and retention mirrors broader trends in nursing workforce research, suggesting that the challenges identified here reflect systemic issues rather than isolated phenomena.

Future research should build on these findings by employing longitudinal designs to examine changes in job satisfaction and turnover intention over time among military nurses. Comparative studies involving civilian and military nursing populations could further clarify the extent to which observed patterns are context-specific or universal. In addition, qualitative research exploring nurses' perceptions of leadership, career progression, and organizational constraints across different service lines may provide deeper insight into the mechanisms underlying satisfaction and retention in military healthcare settings.

### 4.2 Perspectives for clinical and assistive practice

The findings of this study extend beyond human resources management and have direct implications for clinical and assistive practice within the Hellenic Navy. The high dissatisfaction with extrinsic factors (pay, promotion, operating conditions) and their association with turnover intention is not solely an organizational challenge; it represents a direct risk to patient care continuity and quality. Experienced nurses carry valuable clinical knowledge and technical skill, and their attrition results in the loss of institutional expertise that is not easily replaced. Moreover, persistent dissatisfaction and stress arising from inadequate working conditions and insufficient rewards are well-established precursors to burnout (39, 40). Contemporary research demonstrates that burnout among healthcare workers is widespread and is directly linked to depression, diminished quality of life, and reductions in care quality (41). For military nurses, who already operate under unique psychological and operational stressors, these organizational shortcomings can heighten burnout risk, contributing to detachment from patients and diminished capacity for high-quality clinical and assistive care.

Thus, improving leadership, communication, and organizational rewards is fundamentally an effort to enhance the clinical environment. Supportive leaders who buffer stress, foster psychological safety, and advocate for their teams can meaningfully reduce burnout risk (17). From an assistive practice perspective, our findings highlight the necessity of ensuring that Navy nurses are adequately supported in performing both clinical and assistive care tasks efficiently and safely. Strengthening communication pathways, optimizing workload distribution, and reinforcing supervisory support directly enhance nurses' capacity to deliver high-quality bedside and assistive care, especially in high-pressure military settings. Addressing the extrinsic dissatisfiers identified in this study is essential for cultivating a work environment in which nurses can thrive professionally and personally, ensuring they remain psychologically and physically prepared to provide optimal care to service members and their families. Retention strategies should therefore be framed not only as workforce initiatives but as critical investments in clinical excellence and patient safety.

Overall, these clinical and assistive implications underscore that leadership development and organizational improvements are not merely staffing considerations but essential components of maintaining operational readiness, continuity of care, and patient safety within the Hellenic Navy.

### 4.3 Limitations

Despite these contributions, this study has several limitations. First, our research focuses exclusively on officer nurses who graduated from the SAN. Therefore, the findings may not be generalizable to other nursing personnel within the Hellenic Navy who may have different educational backgrounds or ranks. This specificity of the sample, while providing deep insights into a key demographic, restricts the conclusions to this particular cohort. Data were also collected via self-administered questionnaires, which introduces the possibility of social desirability bias; participants may have reported intentions that aligned with socially acceptable norms. Although anonymity and voluntary participation were ensured, the potential for inaccurate responses cannot be completely ruled out. In addition, the cross-sectional design captures attitudes and intentions at a single point in time, precluding causal inferences regarding the relationship between job satisfaction and turnover intention. Future research should employ longitudinal designs and larger, more diverse samples to better explore causal pathways and the long-term impact of organizational interventions on nurse retention in military settings.

## 5 Conclusion

This study demonstrates that job satisfaction and turnover intention among Greek Navy nurses are shaped by a combination of intrinsic and extrinsic factors, with leadership—particularly supportive supervision and effective communication—playing a central role. While interpersonal aspects of work emerged as key sources of satisfaction, dissatisfaction with organizational and structural factors such as pay, promotion opportunities, workload, and fringe benefits was prominent and closely linked to turnover intention.

These findings underscore the importance of leadership development and organizational support in military healthcare settings. Interventions aimed at strengthening supervisory practices, improving communication, and addressing extrinsic sources of dissatisfaction may enhance job satisfaction and contribute to workforce stability. By targeting both leadership and organizational conditions, military healthcare organizations can reduce turnover intention and support the retention of a motivated and effective nursing workforce.

## Data Availability

The raw data supporting the conclusions of this article will be made available by the authors, without undue reservation.
