# Supplementary material for: The role of leadership in job satisfaction and turnover intention among Navy nurses: a cross-sectional study in Greece
Source: Front Health Serv. 2026 Jan 16;5:1712270. doi: 10.3389/frhs.2025.1712270 (PMC12855411; doi:10.3389/frhs.2025.1712270)
Supplement: Supplementary file 1 [file Table1.docx]

Supplementary Material

**Supplementary Table 1.** STROBE checklist for cross-sectional studies.

|  | Item No | Recommendation | Section and quoted text |
| --- | --- | --- | --- |
| **Title and abstract** | 1 | (*a*) Indicate the study’s design with a commonly used term in the title or the abstract | Title**:** “*… a cross-sectional study in Greece*”  Abstract: “*Methods: A cross-sectional survey was conducted…*” |
|  |  | (*b*) Provide in the abstract an informative and balanced summary of what was done and what was found | Abstract: “*Methods: A cross-sectional survey was conducted from January to March 2025 … Analyses employed descriptive statistics and bivariate tests.*” and “*Results: While respondents reported satisfaction with supervision (68.3%) … significantly correlated with turnover intentions.*” |
| Introduction | | | |
| Background/  rationale | 2 | Explain the scientific background and rationale for the investigation being reported | 1. Introduction: “*Job satisfaction is a multifaceted construct … factors that promote satisfaction and retention (4).*” and “*These challenges are particularly salient in military healthcare settings … Accordingly, this study aims to address this gap.*” |
| Objectives | 3 | State specific objectives, including any prespecified hypotheses | 1. Introduction: “*The primary objective was to investigate levels of job satisfaction and turnover intention among Greek Navy nurses. Secondary objectives were to explore the relationships between specific facets of job satisfaction and turnover intention and to examine the influence of leadership—particularly supervision—on these outcomes.*” |
| Methods | | | |
| Study design | 4 | Present key elements of study design early in the paper | 2.1 Study design and reporting: “*This cross-sectional study was designed and reported … a completed checklist is provided as Supplementary Table 1*” |
| Setting | 5 | Describe the setting, locations, and relevant dates, including periods of recruitment, exposure, follow-up, and data collection | 2.1 Study design and reporting: “*Data collection took place between January and March 2025, with all data obtained at a single time point; as a cross-sectional design was used, there was no exposure period or follow-up.*”  2.2 Study setting: “*The study was conducted at the NNA … due to organizational confidentiality restrictions.*” |
| Participants | 6 | (*a*) Give the eligibility criteria, and the sources and methods of selection of participants | 2.3 Participants: “*All active-duty Navy nurse officers … within this specific upper-level professional cohort.*” |
| Variables | 7 | Clearly define all outcomes, exposures, predictors, potential confounders, and effect modifiers. Give diagnostic criteria, if applicable | 2.4 Data collection and instruments:  Outcomes**:** “*Job satisfaction was measured with the 36-item Job Satisfaction Survey (JSS) … also rated on a 6-point Likert scale for consistency.*”  Exposures / Predictors: “*Predictor variables included demographic and professional characteristics (age, gender, marital status, number of children, education level, rank, and years of service) and perceived leadership quality (JSS supervision subscale)*”  Potential confounders and effect modifiers: “*Because the study was descriptive, no confounders or effect modifiers were modeled.*”  Diagnostic criteria: Not applicable. |
| Data sources/ measurement | 8* | For each variable of interest, give sources of data and details of methods of assessment (measurement). Describe comparability of assessment methods if there is more than one group | 2.4 Data collection and instruments:  For job satisfaction: “*Job satisfaction was measured with the 36-item Job Satisfaction Survey (JSS) … Reverse-scored items were recoded before computing domain and total scores.*”  For turnover intention: “*Turnover intention was assessed using items* … also rated on a 6-point Likert scale for consistency.”  For demographic and professional variables: “*Predictor variables included demographic and professional characteristics (age, gender, marital status, number of children, education level, rank, and years of service)*”  Comparability of Assessment Methods Across Groups: “*All participants received the same instruments and instructions, ensuring full comparability across subgroups.*” |
| Bias | 9 | Describe any efforts to address potential sources of bias | 2.5 Bias and study size: “*Multiple procedures minimized potential bias. To reduce selection bias, all eligible nurses were invited to participate regardless of shift. To reduce information and social desirability bias, questionnaires were anonymous and confidentiality was emphasized. Measurement bias was minimized by administering identical instruments under uniform conditions. Because there was no follow-up, attrition bias was not applicable.*” |
| Study size | 10 | Explain how the study size was arrived at | 2.5 Bias and study size: “*Because the population was finite and clearly defined, and the study exploratory in nature, no a priori power calculation was performed and a census-like sampling approach was adopted.*” |
| Quantitative variables | 11 | Explain how quantitative variables were handled in the analyses. If applicable, describe which groupings were chosen and why | 2.6 Statistical analysis: “*Quantitative variables were treated as continuous … associations between satisfaction dimensions and turnover intention*” |
| Statistical methods | 12 | (*a*) Describe all statistical methods, including those used to control for confounding | 2.6 Statistical analysis: “*Quantitative variables were treated as continuous … associations between satisfaction dimensions and turnover intention*” |
|  |  | (*b*) Describe any methods used to examine subgroups and interactions | 2.6 Statistical analysis: “*Group comparisons were performed using t-tests for two independent groups and one-way ANOVA for comparisons across multiple demographic subgroups.*” |
|  |  | (*c*) Explain how missing data were addressed | 2.6 Statistical analysis: “*In the event of missing data, analyses were planned to use pairwise deletion, whereby cases with missing responses on specific items would be excluded only from the relevant analyses.*” |
|  |  | (*d*) If applicable, describe analytical methods taking account of sampling strategy | 2.5 Bias and study size: “*Because the population was finite and clearly defined, and the study exploratory in nature, no a priori power calculation was performed and a census-like sampling approach was adopted.*” |
|  |  | (*e*) Describe any sensitivity analyses | Not applicable |
| Results | | | |
| Participants | 13* | (a) Report numbers of individuals at each stage of study—eg numbers potentially eligible, examined for eligibility, confirmed eligible, included in the study, completing follow-up, and analysed | 3. Results: “*Of the 112 eligible active-duty Navy nurse officers invited to participate, 60 returned completed questionnaires, yielding a response rate of 53.6%.*” |
|  |  | (b) Give reasons for non-participation at each stage | 2.3 Participants: “*Nurses who declined participation typically did so due to workload constraints and long-term leave.*” |
|  |  | (c) Consider use of a flow diagram | Not included due to restrictions on the number of figures (for this article type) |
| Descriptive data | 14* | (a) Give characteristics of study participants (eg demographic, clinical, social) and information on exposures and potential confounders | 3. Results: Table 1 and its corresponding interpretation. |
|  |  | (b) Indicate number of participants with missing data for each variable of interest | Not applicable. 3. Results: “*All returned questionnaires were complete, and no missing data were identified.*” |
| Outcome data | 15* | Report numbers of outcome events or summary measures | 3. Results: Tables 2-4 and their corresponding interpretation. |
| Main results | 16 | (*a*) Give unadjusted estimates and, if applicable, confounder-adjusted estimates and their precision (eg, 95% confidence interval). Make clear which confounders were adjusted for and why they were included | Not applicable (no adjusted analyses were conducted, as the aim of the study was descriptive and exploratory, and analyses were limited to descriptive measures and bivariate correlations) |
|  |  | (*b*) Report category boundaries when continuous variables were categorized | Not applicable. No continuous variables were categorized; all continuous variables (e.g., age, JSS subscale scores) were analyzed in their original continuous form. |
|  |  | (*c*) If relevant, consider translating estimates of relative risk into absolute risk for a meaningful time period | Not applicable. The study did not estimate risks or relative risk measures; analyses were limited to descriptive statistics and bivariate correlations. |
| Other analyses | 17 | Report other analyses done—eg analyses of subgroups and interactions, and sensitivity analyses | Not applicable. No subgroup, interaction, or sensitivity analyses were conducted, as the study was descriptive and based on the entire available Navy nursing population. |
| Discussion | | | |
| Key results | 18 | Summarise key results with reference to study objectives | 4.1 Overview of key findings: “*The findings of this study highlight a clear … aimed at specific demographic groups (36)*” |
| Limitations | 19 | Discuss limitations of the study, taking into account sources of potential bias or imprecision. Discuss both direction and magnitude of any potential bias | 4.3 Limitations |
| Interpretation | 20 | Give a cautious overall interpretation of results considering objectives, limitations, multiplicity of analyses, results from similar studies, and other relevant evidence | 4.1 Overview of key findings: “*Overall, the results indicate that Greek Navy nurses … observed in similar studies internationally.*” |
| Generalisability | 21 | Discuss the generalisability (external validity) of the study results | 4.3 Limitations: “*First, our research focuses exclusively on officer nurses … restricts the conclusions to this particular cohort.*” |
| Other information | | | |
| Funding | 22 | Give the source of funding and the role of the funders for the present study and, if applicable, for the original study on which the present article is based | Funding statement: “*The authors declare that no financial support was received for the research, authorship, and/or publication of this article.*” |

**Supplementary Table 2.** Independent-samples t-test by gender.

|  | | Levene's Test for Equality of Variances | | t-test for Equality of Means | | | | | | | |
| --- | --- | --- | --- | --- | --- | --- | --- | --- | --- | --- | --- |
|  |  | F | Sig. | t | df | Significance | | Mean Difference | Std. Error Difference | 95% Confidence Interval of the Difference | |
|  |  |  |  |  |  | One-Sided p | Two-Sided p |  |  | Lower | Upper |
| S10 | Equal variances assumed | 0.006 | 0.940 | 0.562 | 58 | 0.288 | 0.576 | 3.954 | 7.038 | -10.134 | 18.043 |
|  | Equal variances not assumed |  |  | 0.578 | 11.285 | 0.287 | 0.575 | 3.954 | 6.840 | -11.055 | 18.964 |
| T1 | Equal variances assumed | 0.094 | 0.760 | 0.423 | 58 | 0.337 | 0.674 | 0.294 | 0.695 | -1.098 | 1.686 |
|  | Equal variances not assumed |  |  | 0.399 | 10.542 | 0.349 | 0.698 | 0.294 | 0.737 | -1.336 | 1.925 |
| T2 | Equal variances assumed | 5.072 | 0.028 | -1.254 | 58 | 0.107 | 0.215 | -0.699 | 0.558 | -1.815 | 0.417 |
|  | Equal variances not assumed |  |  | -0.949 | 9.309 | 0.183 | 0.367 | -0.699 | 0.737 | -2.358 | 0.959 |
| T3 | Equal variances assumed | 0.818 | 0.370 | -1.077 | 58 | 0.143 | 0.286 | -0.471 | 0.437 | -1.345 | 0.404 |
|  | Equal variances not assumed |  |  | -0.998 | 10.410 | 0.170 | 0.341 | -0.471 | 0.471 | -1.515 | 0.574 |

**Supplementary Table 3.** One-way ANOVA results by age groups.

|  | | Sum of Squares | df | Mean Square | F | Sig. |
| --- | --- | --- | --- | --- | --- | --- |
| S10 | Between Groups | 468.317 | 2 | 234.158 | 0.617 | 0.543 |
|  | Within Groups | 21630.267 | 57 | 379.478 |  |  |
|  | Total | 22098.583 | 59 |  |  |  |
| T1 | Between Groups | 50.855 | 2 | 25.427 | 0.855 | 0.430 |
|  | Within Groups | 164.395 | 57 | 2.884 |  |  |
|  | Total | 215.250 | 59 |  |  |  |
| T2 | Between Groups | 10.868 | 2 | 5.434 | 2.368 | 0.103 |
|  | Within Groups | 130.782 | 57 | 2.294 |  |  |
|  | Total | 141.650 | 59 |  |  |  |
| T3 | Between Groups | 32.606 | 2 | 16.303 | 1.530 | 0.226 |
|  | Within Groups | 53.794 | 57 | 0.944 |  |  |
|  | Total | 86.400 | 59 |  |  |  |

**Supplementary Table 4.** Independent-samples t-test by marital status.

|  | | Levene's Test for Equality of Variances | | t-test for Equality of Means | | | | | | | |
| --- | --- | --- | --- | --- | --- | --- | --- | --- | --- | --- | --- |
|  |  | F | Sig. | t | df | Significance | | Mean Difference | Std. Error Difference | 95% Confidence Interval of the Difference | |
|  |  |  |  |  |  | One-Sided p | Two-Sided p |  |  | Lower | Upper |
| S10 | Equal variances assumed | 0.068 | 0.796 | 0.519 | 58 | 0.303 | 0.606 | 2.685 | 5.171 | -7.666 | 13.036 |
|  | Equal variances not assumed |  |  | 0.518 | 46.568 | 0.303 | 0.607 | 2.685 | 5.179 | -7.736 | 13.106 |
| T1 | Equal variances assumed | 3.200 | 0.079 | 0.380 | 58 | 0.353 | 0.706 | 0.194 | 0.511 | -0.829 | 1.217 |
|  | Equal variances not assumed |  |  | 0.395 | 52.825 | 0.347 | 0.694 | 0.194 | 0.490 | -0.790 | 1.177 |
| T2 | Equal variances assumed | 8.543 | 0.005 | 0.093 | 58 | 0.463 | 0.926 | 0.039 | 0.415 | -0.792 | 0.869 |
|  | Equal variances not assumed |  |  | 0.102 | 57.432 | 0.460 | 0.919 | 0.039 | 0.380 | -0.723 | 0.800 |
| T3 | Equal variances assumed | 9.356 | 0.003 | -2.074 | 58 | 0.041 | 0.082 | -0.649 | 0.313 | -1.275 | -0.023 |
|  | Equal variances not assumed |  |  | -1.961 | 38.656 | 0.033 | 0.066 | -0.649 | 0.331 | -1.318 | 0.021 |

**Supplementary Table 5.** One-way ANOVA results by employer.

|  | | Sum of Squares | df | Mean Square | F | Sig. |
| --- | --- | --- | --- | --- | --- | --- |
| S10 | Between Groups | 618.967 | 3 | 206.322 | 0.538 | 0.658 |
|  | Within Groups | 21479.617 | 56 | 383.565 |  |  |
|  | Total | 22098.583 | 59 |  |  |  |
| T1 | Between Groups | 40.295 | 3 | 13.432 | 1.299 | 0.808 |
|  | Within Groups | 174.955 | 56 | 3.124 |  |  |
|  | Total | 215.250 | 59 |  |  |  |
| T2 | Between Groups | 7.410 | 3 | 2.470 | 1.030 | 0.386 |
|  | Within Groups | 134.240 | 56 | 2.397 |  |  |
|  | Total | 141.650 | 59 |  |  |  |
| T3 | Between Groups | 3.655 | 3 | 1.218 | 0.824 | 0.486 |
|  | Within Groups | 82.745 | 56 | 1.478 |  |  |
|  | Total | 86.400 | 59 |  |  |  |

**Supplementary Table 6.** One-way ANOVA results by work department.

|  | | Sum of Squares | df | Mean Square | F | Sig. |
| --- | --- | --- | --- | --- | --- | --- |
| S10 | Between Groups | 750.884 | 4 | 187.721 | 0.484 | 0.748 |
|  | Within Groups | 21347.699 | 55 | 388.140 |  |  |
|  | Total | 22098.583 | 59 |  |  |  |
| T1 | Between Groups | 3.396 | 4 | 0.849 | 0.220 | 0.926 |
|  | Within Groups | 211.854 | 55 | 3.852 |  |  |
|  | Total | 215.250 | 59 |  |  |  |
| T2 | Between Groups | 5.790 | 4 | 1.447 | 0.586 | 0.674 |
|  | Within Groups | 135.860 | 55 | 2.470 |  |  |
|  | Total | 141.650 | 59 |  |  |  |
| T3 | Between Groups | 3.482 | 4 | 0.871 | 0.577 | 0.680 |
|  | Within Groups | 82.918 | 55 | 1.508 |  |  |
|  | Total | 86.400 | 59 |  |  |  |

**Supplementary Table 7.** One-way ANOVA results by years of service.

|  | | Sum of Squares | df | Mean Square | F | Sig. |
| --- | --- | --- | --- | --- | --- | --- |
| S10 | Between Groups | 1409.520 | 6 | 234.920 | 0.602 | 0.728 |
|  | Within Groups | 20689.063 | 53 | 390.360 |  |  |
|  | Total | 22098.583 | 59 |  |  |  |
| T1 | Between Groups | 78.268 | 6 | 13.045 | 1.047 | 0.505 |
|  | Within Groups | 136.982 | 53 | 2.585 |  |  |
|  | Total | 215.250 | 59 |  |  |  |
| T2 | Between Groups | 31.241 | 6 | 5.207 | 1.872 | 0.075 |
|  | Within Groups | 110.409 | 53 | 2.083 |  |  |
|  | Total | 141.650 | 59 |  |  |  |
| T3 | Between Groups | 30.278 | 6 | 5.046 | 1.766 | 0.088 |
|  | Within Groups | 56.122 | 53 | 1.059 |  |  |
|  | Total | 86.400 | 59 |  |  |  |

**Supplementary Table 8.** One-way ANOVA results by work schedule.

|  | | Sum of Squares | df | Mean Square | F | Sig. |
| --- | --- | --- | --- | --- | --- | --- |
| S10 | Between Groups | 379.900 | 2 | 189.950 | 0.499 | 0.610 |
|  | Within Groups | 21718.683 | 57 | 381.030 |  |  |
|  | Total | 22098.583 | 59 |  |  |  |
| T1 | Between Groups | 17.861 | 2 | 8.931 | 2.579 | 0.085 |
|  | Within Groups | 197.389 | 57 | 3.463 |  |  |
|  | Total | 215.250 | 59 |  |  |  |
| T2 | Between Groups | 19.045 | 2 | 9.523 | 2.427 | 0.616 |
|  | Within Groups | 122.605 | 57 | 2.151 |  |  |
|  | Total | 141.650 | 59 |  |  |  |
| T3 | Between Groups | 4.423 | 2 | 2.211 | 1.538 | 0.224 |
|  | Within Groups | 81.977 | 57 | 1.438 |  |  |
|  | Total | 86.400 | 59 |  |  |  |

**Supplementary Table 9.** One-way ANOVA results by education.

|  | | Sum of Squares | df | Mean Square | F | Sig. |
| --- | --- | --- | --- | --- | --- | --- |
| S10 | Between Groups | 1216.208 | 2 | 608.104 | 1.660 | 0.199 |
|  | Within Groups | 20882.375 | 57 | 366.357 |  |  |
|  | Total | 22098.583 | 59 |  |  |  |
| T1 | Between Groups | 19.029 | 2 | 9.514 | 2.764 | 0.072 |
|  | Within Groups | 196.221 | 57 | 3.442 |  |  |
|  | Total | 215.250 | 59 |  |  |  |
| T2 | Between Groups | 4.121 | 2 | 2.061 | 0.854 | 0.431 |
|  | Within Groups | 137.529 | 57 | 2.413 |  |  |
|  | Total | 141.650 | 59 |  |  |  |
| T3 | Between Groups | 2.585 | 2 | 1.293 | 0.879 | 0.421 |
|  | Within Groups | 83.815 | 57 | 1.470 |  |  |
|  | Total | 86.400 | 59 |  |  |  |
